# 4-Methyl-9-[(4-methyl­phen­yl)sulfon­yl]thio­pyrano[3,4-*b*]indole-3(9*H*)-thione

**DOI:** 10.1107/S1600536810038201

**Published:** 2010-09-30

**Authors:** Benjamin Dassonneville, Dieter Schollmeyer, Bernhard Witulski, Heiner Detert

**Affiliations:** a University Mainz, Duesbergweg 10-14, 55099 Mainz, Germany; b Laboratoire de Chimie Moléculaire et Thio-organique, UMR 6507, ENSICAEN, 6 Boulevard Maréchal Juin, 14050 Caen, France

## Abstract

The title compound, C_19_H_15_NO_2_S_3_, is the first example of a dithia analogue of pyrano[3,4-*b*]indolone. The almost planar thio­pyrano­indole­thione ring system (r.m.s. deviation for all non-H atoms = 0.030 Å) makes a dihedral angle of 80.70 (8)° with the *p*-tolyl ring. In the crystal, mol­ecules are connected *via* C—H⋯O hydrogen bonds into two chains along the *b* axis. These chains are connected *via* π–π inter­actions between symmetry-related thio­pyrano­indole­thione ring systems [centroid–centroid distance = 3.588 (1) Å].

## Related literature

The title compound was synthesized as part of a larger project focusing on metal-catalysed transformations of tethered alkynyl-ynamides to carbazoles (Witulski & Alayrac, 2002 ▶) and to carbolines and other heteroannulated indoles (Nissen, 2008 ▶; Dassonneville, 2010 ▶). The reactivity of such an annulated thio­pyran­othione could be similar to the respective pyrano[3,4-*b*]indolone, well known as stable equivalents of indoloquinodimethanes (Plieninger *et al.*, 1964 ▶) and valuable inter­mediates for the synthesis of various heteroannulated indoles, see, for example: Livadiotou *et al.* (2009 ▶).
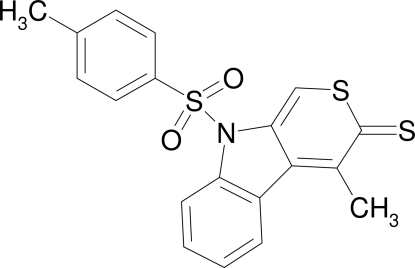


## Experimental

### 

#### Crystal data

C_19_H_15_NO_2_S_3_
*M* *_r_* = 385.50
Monoclinic, 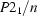

*a* = 13.2530 (4) Å
*b* = 8.2423 (3) Å
*c* = 15.4500 (16) Å
β = 96.124 (3)°
*V* = 1678.05 (19) Å^3^
*Z* = 4
Cu *K*α radiation
μ = 4.15 mm^−1^
*T* = 193 K
0.60 × 0.10 × 0.10 mm

#### Data collection

Enraf–Nonius CAD-4 diffractometer
Absorption correction: numerical (de Meulenaer & Tompa, 1965 ▶) *T*_min_ = 0.42, *T*_max_ = 0.70
3167 measured reflections
3167 independent reflections
2714 reflections with *I* > 2σ(*I*)
3 standard reflections every 60 min  intensity decay: 2%

#### Refinement

*R*[*F* ^2^ > 2σ(*F* ^2^)] = 0.037
*wR*(*F* ^2^) = 0.102
*S* = 1.04
3167 reflections
228 parameters
H-atom parameters constrained
Δρ_max_ = 0.42 e Å^−3^
Δρ_min_ = −0.36 e Å^−3^

### 

Data collection: *CAD-4 Software* (Enraf–Nonius, 1989 ▶); cell refinement: *CAD-4 Software*; data reduction: *CORINC* (Dräger & Gattow, 1971 ▶); program(s) used to solve structure: *SIR97* (Altomare *et al.*, 1999 ▶); program(s) used to refine structure: *SHELXL97* (Sheldrick, 2008 ▶); molecular graphics: *PLATON* (Spek, 2009 ▶); software used to prepare material for publication: *PLATON*.

## Supplementary Material

Crystal structure: contains datablocks I, global. DOI: 10.1107/S1600536810038201/bt5362sup1.cif

Structure factors: contains datablocks I. DOI: 10.1107/S1600536810038201/bt5362Isup2.hkl

Additional supplementary materials:  crystallographic information; 3D view; checkCIF report

## Figures and Tables

**Table 1:** Hydrogen-bond geometry (Å, °)

*D*—H⋯*A*	*D*—H	H⋯*A*	*D*⋯*A*	*D*—H⋯*A*
C21—H21⋯O15^i^	0.95	2.50	3.387 (3)	155
C22—H22⋯O16^ii^	0.95	2.59	3.116 (3)	116

Symmetry codes: (i) 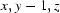; (ii) 
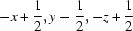
.

**Fig. 1.**
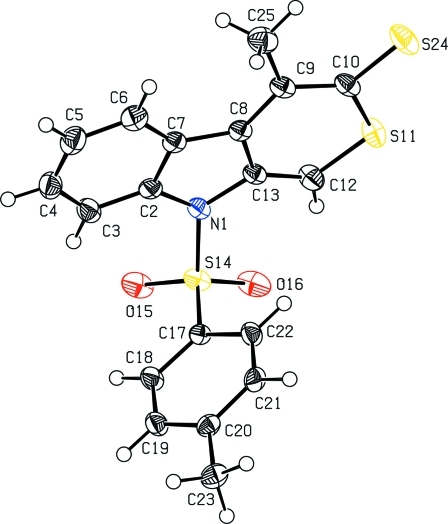
Perspective view the title compound. Displacement ellipsoids are drawn at the 50% probability level.

**Fig. 2.**
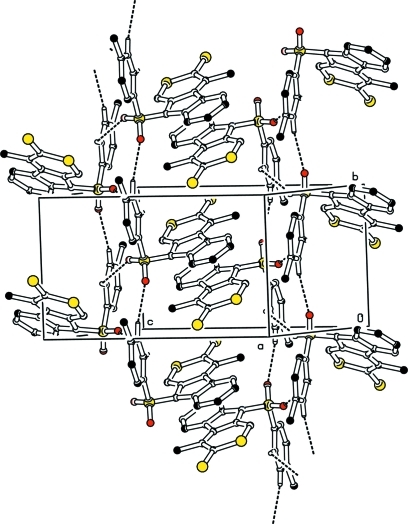
Part of the packing diagram showing the hydrogen bonds and the π-π-interactions.

## supplementary crystallographic information

### Comment

The title compound is formed *via* a rhodium-catalyzed [2 + 2+2] cycloaddition of a tethered alkynyl-ynamide and carbon disulfide. This unprecedented thiopyranoindolethione is obtained as dark violet crystals. The crystal structure of the title compound forms a network built by two chains along the *b* axis connected *via* hydrogen bonds C21—H21···O15 (2.50 Å) and C22—H21···O16 (2.59 Å). These chains are connected *via*π-π-interactions of two thiopyranoindolethiones related by a center of symmetry [distance between centroids 3.588 (1) Å].

The tricyclic framework is essentially planar with torsion angles of about 2.1 ° or less in the benzene moiety and up to 5.9 ° in the thiopyrane unit. The torsion angle around the biphenyl bond amounts to 176.0 (2) ° (C6,C7,C8,C13). Whereas the N—C bonds in the pyrrole ring are nearly identical, the C—C bond lengths increase in the sequence C2—C7, C7—C8, C8—C13. With 1.459 (3) Å, the biphenyl bond C7—C8 is significantly longer than C2—C3 (1.384 (3) Å) and C8—C13 (1.443 (3) Å). The thiocarbonyl bond length is about 1.668 (2) Å, much shorter than the C—S single bonds with 1.729 (2) Å (C10—S11) and 1.702 (2) Å (S11—C12). Due to steric repulsion of methyl and thiocarbonyl, the S24—C10—S11 bond angle is reduced to 113.07 (13) ° and the C10—S11—C12 bond angle is only 107.07 (11) °.

### Experimental

The title compound was prepared from 2-(propynyl)-*N*-ethynyl-*N*-[(4-methylphenyl)sulfonyl)]benzenamine (Witulski & Alayrac, 2002)) as follows: Under Argon (Ar), BINAP (15.6 mg, 0.025 mmol, 10 mol%) and [RhCl(C_8_H_14_)_2_]_2_ (6.1 mg, 0.0087 mmol, 3.5 mol%) are dissolved in degassed CH_2_Cl_2_ (3.0 ml) in a Schlenk tube, and the mixture is stirred at room temperature for 5 min. H_2_ is then introduced to the resulting solution. After stirring at room temperature for 0.5 h, the resulting solution is concentrated to dryness and the residue dissolved in dichloroethane (DCE) (3.0 ml). To this solution is added dropwise over 1 min a solution of the alkynyl-ynamide (0.25 mmol) and CS_2_ (150 µ*L*, 2.5 mmol, 10 equiv.) in DCE (5.0 ml). Undissolved substrate is dissolved by addition of DCE (2x1.0 ml), added to the solution and the mixture is heated to 353 K. After completion of the reaction (3 h, TLC), the solvent is removed and the residue is purified by column chromatography (Al_2_O_3_, Petroleum ether/Ethyl acetate, 95/5). Violet crystals of the title compound suitable for X-ray analysis were obtained by crystallization from CH_2_Cl_2_/Petroleum ether.

### Refinement

Hydrogen atoms  were placed at calculated positions with C—H = 0.95 Å (aromatic) or 0.98 Å (methyl groups). All H atoms were refined in the riding-model approximation with isotropic displacement parameters (set at 1.2–1.5 times of the *U*_eq_ of the parent atom).

### Crystal data

**Table:**

C_19_H_15_NO_2_S_3_	*F*(000) = 800
*M**_r_* = 385.50	*D*_x_ = 1.526 Mg m^−^^3^
Monoclinic, *P*2_1_/*n*	Cu *K*α radiation, λ = 1.54178 Å
Hall symbol: -P 2yn	Cell parameters from 25 reflections
*a* = 13.2530 (4) Å	θ = 60–70°
*b* = 8.2423 (3) Å	µ = 4.15 mm^−^^1^
*c* = 15.4500 (16) Å	*T* = 193 K
β = 96.124 (3)°	Needle, violet
*V* = 1678.05 (19) Å^3^	0.60 × 0.10 × 0.10 mm
*Z* = 4	

### Data collection

**Table:**

Enraf–Nonius CAD-4 diffractometer	2714 reflections with *I* > 2σ(*I*)
Radiation source: rotating anode	*R*_int_ = 0.0000
graphite	θ_max_ = 69.9°, θ_min_ = 4.2°
ω/2θ scans	*h* = 0→16
Absorption correction: numerical (de Meulenaer & Tompa, 1965)	*k* = 0→10
*T*_min_ = 0.42, *T*_max_ = 0.70	*l* = −18→18
3167 measured reflections	3 standard reflections every 60 min
3167 independent reflections	intensity decay: 2%

### Refinement

**Table:**

Refinement on *F*^2^	Primary atom site location: structure-invariant direct methods
Least-squares matrix: full	Secondary atom site location: difference Fourier map
*R*[*F*^2^ > 2σ(*F*^2^)] = 0.037	Hydrogen site location: inferred from neighbouring sites
*wR*(*F*^2^) = 0.102	H-atom parameters constrained
*S* = 1.04	*w* = 1/[σ^2^(*F*_o_^2^) + (0.053*P*)^2^ + 0.8161*P*] where *P* = (*F*_o_^2^ + 2*F*_c_^2^)/3
3167 reflections	(Δ/σ)_max_ < 0.001
228 parameters	Δρ_max_ = 0.42 e Å^−^^3^
0 restraints	Δρ_min_ = −0.36 e Å^−^^3^

### Special details

**Table:**

Geometry. All e.s.d.'s (except the e.s.d. in the dihedral angle between two l.s. planes) are estimated using the full covariance matrix. The cell e.s.d.'s are taken into account individually in the estimation of e.s.d.'s in distances, angles and torsion angles; correlations between e.s.d.'s in cell parameters are only used when they are defined by crystal symmetry. An approximate (isotropic) treatment of cell e.s.d.'s is used for estimating e.s.d.'s involving l.s. planes.
Refinement. Refinement of *F*^2^ against ALL reflections. The weighted *R*-factor *wR* and goodness of fit *S* are based on *F*^2^, conventional *R*-factors *R* are based on *F*, with *F* set to zero for negative *F*^2^. The threshold expression of *F*^2^ > σ(*F*^2^) is used only for calculating *R*-factors(gt) *etc*. and is not relevant to the choice of reflections for refinement. *R*-factors based on *F*^2^ are statistically about twice as large as those based on *F*, and *R*- factors based on ALL data will be even larger.

### Fractional atomic coordinates and isotropic or equivalent isotropic displacement parameters (Å^2^)

**Table:**

	*x*	*y*	*z*	*U*_iso_*/*U*_eq_	
N1	0.30393 (13)	0.4410 (2)	0.44134 (12)	0.0228 (4)	
C2	0.29369 (15)	0.4317 (3)	0.53192 (14)	0.0230 (4)	
C3	0.21571 (17)	0.4930 (3)	0.57513 (16)	0.0293 (5)	
H3	0.1611	0.5518	0.5451	0.035*	
C4	0.22049 (18)	0.4654 (3)	0.66365 (16)	0.0336 (5)	
H4	0.1683	0.5064	0.6952	0.040*	
C5	0.30000 (19)	0.3790 (3)	0.70717 (16)	0.0346 (5)	
H5	0.3005	0.3596	0.7678	0.042*	
C6	0.37865 (18)	0.3204 (3)	0.66405 (15)	0.0309 (5)	
H6	0.4333	0.2625	0.6947	0.037*	
C7	0.37639 (15)	0.3478 (2)	0.57428 (14)	0.0222 (4)	
C8	0.44351 (15)	0.3017 (2)	0.50942 (14)	0.0211 (4)	
C9	0.53663 (16)	0.2244 (3)	0.52013 (15)	0.0243 (4)	
C10	0.58968 (16)	0.1801 (3)	0.44741 (16)	0.0263 (5)	
S11	0.54433 (4)	0.23832 (8)	0.34297 (4)	0.03407 (17)	
C12	0.43366 (17)	0.3397 (3)	0.34993 (15)	0.0303 (5)	
H12	0.3984	0.3847	0.2987	0.036*	
C13	0.39482 (15)	0.3576 (2)	0.42680 (14)	0.0217 (4)	
S14	0.20305 (4)	0.46546 (6)	0.36740 (4)	0.02438 (15)	
O15	0.15035 (12)	0.60556 (19)	0.39288 (12)	0.0353 (4)	
O16	0.24145 (12)	0.4613 (2)	0.28475 (11)	0.0367 (4)	
C17	0.12629 (15)	0.2950 (2)	0.37845 (13)	0.0194 (4)	
C18	0.02728 (16)	0.3153 (3)	0.39707 (15)	0.0268 (5)	
H18	0.0018	0.4205	0.4072	0.032*	
C19	−0.03435 (16)	0.1799 (3)	0.40080 (15)	0.0276 (5)	
H19	−0.1023	0.1931	0.4137	0.033*	
C20	0.00193 (16)	0.0253 (3)	0.38587 (14)	0.0238 (4)	
C21	0.10251 (16)	0.0078 (3)	0.36982 (14)	0.0254 (5)	
H21	0.1288	−0.0977	0.3619	0.031*	
C22	0.16513 (16)	0.1414 (3)	0.36515 (14)	0.0252 (5)	
H22	0.2335	0.1283	0.3531	0.030*	
C23	−0.06630 (18)	−0.1206 (3)	0.38685 (16)	0.0301 (5)	
H23A	−0.1058	−0.1136	0.4368	0.045*	
H23B	−0.0250	−0.2195	0.3914	0.045*	
H23C	−0.1125	−0.1236	0.3329	0.045*	
S24	0.69645 (4)	0.07193 (8)	0.45140 (5)	0.04028 (18)	
C25	0.58643 (19)	0.1818 (3)	0.60888 (17)	0.0365 (6)	
H25A	0.5844	0.2758	0.6475	0.055*	
H25B	0.6572	0.1509	0.6048	0.055*	
H25C	0.5503	0.0908	0.6324	0.055*	

### Atomic displacement parameters (Å^2^)

**Table:**

	*U*^11^	*U*^22^	*U*^33^	*U*^12^	*U*^13^	*U*^23^
N1	0.0188 (8)	0.0218 (9)	0.0278 (9)	0.0008 (7)	0.0021 (7)	0.0015 (7)
C2	0.0217 (10)	0.0168 (10)	0.0303 (11)	−0.0057 (8)	0.0021 (8)	−0.0027 (8)
C3	0.0239 (11)	0.0226 (11)	0.0419 (13)	−0.0002 (9)	0.0058 (9)	−0.0042 (10)
C4	0.0323 (12)	0.0306 (13)	0.0397 (13)	−0.0057 (10)	0.0124 (10)	−0.0098 (10)
C5	0.0410 (13)	0.0341 (13)	0.0299 (12)	−0.0074 (11)	0.0089 (10)	−0.0040 (10)
C6	0.0328 (12)	0.0296 (12)	0.0300 (11)	−0.0031 (10)	0.0023 (9)	0.0013 (10)
C7	0.0225 (10)	0.0144 (10)	0.0295 (11)	−0.0048 (8)	0.0021 (8)	−0.0003 (8)
C8	0.0215 (10)	0.0142 (9)	0.0276 (10)	−0.0039 (8)	0.0021 (8)	0.0010 (8)
C9	0.0207 (10)	0.0166 (10)	0.0354 (11)	−0.0020 (8)	0.0014 (8)	0.0027 (9)
C10	0.0192 (10)	0.0168 (10)	0.0435 (13)	−0.0028 (8)	0.0057 (9)	0.0016 (9)
S11	0.0296 (3)	0.0377 (3)	0.0369 (3)	0.0052 (2)	0.0126 (2)	0.0018 (3)
C12	0.0257 (11)	0.0360 (13)	0.0296 (11)	0.0038 (10)	0.0044 (9)	0.0049 (10)
C13	0.0185 (9)	0.0172 (10)	0.0293 (11)	−0.0017 (8)	0.0020 (8)	0.0021 (8)
S14	0.0203 (3)	0.0180 (3)	0.0340 (3)	−0.00007 (19)	−0.0006 (2)	0.0073 (2)
O15	0.0286 (8)	0.0155 (8)	0.0602 (11)	0.0028 (7)	−0.0026 (8)	0.0043 (7)
O16	0.0284 (8)	0.0483 (11)	0.0328 (9)	−0.0043 (8)	0.0003 (7)	0.0169 (8)
C17	0.0197 (9)	0.0135 (10)	0.0245 (10)	0.0000 (8)	0.0005 (8)	0.0010 (8)
C18	0.0218 (10)	0.0175 (11)	0.0408 (12)	0.0042 (8)	0.0027 (9)	−0.0034 (9)
C19	0.0217 (10)	0.0225 (11)	0.0394 (12)	0.0003 (9)	0.0066 (9)	−0.0045 (9)
C20	0.0273 (11)	0.0193 (11)	0.0241 (10)	−0.0005 (9)	−0.0006 (8)	−0.0012 (8)
C21	0.0295 (11)	0.0158 (10)	0.0305 (11)	0.0054 (9)	0.0008 (9)	−0.0043 (9)
C22	0.0224 (10)	0.0233 (11)	0.0301 (11)	0.0047 (9)	0.0045 (8)	−0.0014 (9)
C23	0.0346 (12)	0.0206 (11)	0.0345 (12)	−0.0052 (9)	0.0011 (9)	−0.0017 (9)
S24	0.0255 (3)	0.0312 (3)	0.0654 (4)	0.0079 (2)	0.0110 (3)	0.0038 (3)
C25	0.0316 (12)	0.0363 (14)	0.0401 (13)	0.0056 (11)	−0.0034 (10)	0.0049 (11)

### Geometric parameters (Å, °)

**Table:**

N1—C2	1.422 (3)	C17—C22	1.390 (3)
N1—C13	1.425 (3)	C18—C19	1.388 (3)
N1—S14	1.6756 (18)	C19—C20	1.390 (3)
C2—C3	1.384 (3)	C20—C21	1.389 (3)
C2—C7	1.398 (3)	C20—C23	1.505 (3)
C3—C4	1.381 (3)	C21—C22	1.385 (3)
C4—C5	1.385 (4)	C3—H3	0.9500
C5—C6	1.383 (3)	C4—H4	0.9500
C6—C7	1.403 (3)	C5—H5	0.9500
C7—C8	1.459 (3)	C6—H6	0.9500
C8—C9	1.383 (3)	C12—H12	0.9500
C8—C13	1.443 (3)	C18—H18	0.9500
C9—C10	1.435 (3)	C19—H19	0.9500
C9—C25	1.499 (3)	C21—H21	0.9500
C10—S24	1.668 (2)	C22—H22	0.9500
C10—S11	1.729 (2)	C23—H23A	0.9800
S11—C12	1.702 (2)	C23—H23B	0.9800
C12—C13	1.352 (3)	C23—H23C	0.9800
S14—O16	1.4245 (18)	C25—H25A	0.9800
S14—O15	1.4265 (17)	C25—H25B	0.9800
S14—C17	1.754 (2)	C25—H25C	0.9800
C17—C18	1.383 (3)		
			
C2—N1—C13	107.46 (17)	C18—C19—C20	121.0 (2)
C2—N1—S14	121.62 (14)	C21—C20—C19	118.7 (2)
C13—N1—S14	125.24 (15)	C21—C20—C23	120.5 (2)
C3—C2—C7	122.9 (2)	C19—C20—C23	120.8 (2)
C3—C2—N1	127.5 (2)	C22—C21—C20	121.2 (2)
C7—C2—N1	109.56 (18)	C21—C22—C17	118.86 (19)
C4—C3—C2	117.4 (2)	C2—C3—H3	121.00
C3—C4—C5	121.2 (2)	C4—C3—H3	121.00
C6—C5—C4	121.3 (2)	C3—C4—H4	119.00
C5—C6—C7	118.8 (2)	C5—C4—H4	119.00
C2—C7—C6	118.4 (2)	C4—C5—H5	119.00
C2—C7—C8	108.18 (18)	C6—C5—H5	119.00
C6—C7—C8	133.4 (2)	C5—C6—H6	121.00
C9—C8—C13	124.2 (2)	C7—C6—H6	121.00
C9—C8—C7	129.7 (2)	S11—C12—H12	119.00
C13—C8—C7	106.06 (17)	C13—C12—H12	119.00
C8—C9—C10	121.9 (2)	C17—C18—H18	120.00
C8—C9—C25	121.2 (2)	C19—C18—H18	120.00
C10—C9—C25	116.88 (19)	C18—C19—H19	119.00
C9—C10—S24	126.28 (18)	C20—C19—H19	120.00
C9—C10—S11	120.65 (16)	C20—C21—H21	119.00
S24—C10—S11	113.07 (13)	C22—C21—H21	119.00
C12—S11—C10	107.07 (11)	C17—C22—H22	121.00
C13—C12—S11	121.37 (18)	C21—C22—H22	121.00
C12—C13—N1	126.8 (2)	C20—C23—H23A	109.00
C12—C13—C8	124.5 (2)	C20—C23—H23B	109.00
N1—C13—C8	108.67 (18)	C20—C23—H23C	109.00
O16—S14—O15	119.95 (11)	H23A—C23—H23B	110.00
O16—S14—N1	105.83 (9)	H23A—C23—H23C	109.00
O15—S14—N1	106.73 (10)	H23B—C23—H23C	109.00
O16—S14—C17	109.53 (10)	C9—C25—H25A	110.00
O15—S14—C17	108.39 (10)	C9—C25—H25B	109.00
N1—S14—C17	105.43 (9)	C9—C25—H25C	109.00
C18—C17—C22	121.02 (19)	H25A—C25—H25B	109.00
C18—C17—S14	119.77 (16)	H25A—C25—H25C	109.00
C22—C17—S14	119.17 (16)	H25B—C25—H25C	109.00
C17—C18—C19	119.2 (2)		
			
C13—N1—C2—C3	−178.6 (2)	S11—C12—C13—C8	3.1 (3)
S14—N1—C2—C3	−24.0 (3)	C2—N1—C13—C12	−178.9 (2)
C13—N1—C2—C7	1.1 (2)	S14—N1—C13—C12	27.6 (3)
S14—N1—C2—C7	155.80 (15)	C2—N1—C13—C8	−2.3 (2)
C7—C2—C3—C4	−1.5 (3)	S14—N1—C13—C8	−155.83 (15)
N1—C2—C3—C4	178.3 (2)	C9—C8—C13—C12	0.7 (3)
C2—C3—C4—C5	−0.3 (3)	C7—C8—C13—C12	179.3 (2)
C3—C4—C5—C6	1.5 (4)	C9—C8—C13—N1	−176.00 (19)
C4—C5—C6—C7	−0.9 (4)	C7—C8—C13—N1	2.6 (2)
C3—C2—C7—C6	2.1 (3)	C2—N1—S14—O16	−177.20 (16)
N1—C2—C7—C6	−177.71 (18)	C13—N1—S14—O16	−27.17 (19)
C3—C2—C7—C8	−179.74 (19)	C2—N1—S14—O15	53.96 (18)
N1—C2—C7—C8	0.5 (2)	C13—N1—S14—O15	−156.01 (17)
C5—C6—C7—C2	−0.8 (3)	C2—N1—S14—C17	−61.18 (18)
C5—C6—C7—C8	−178.5 (2)	C13—N1—S14—C17	88.86 (18)
C2—C7—C8—C9	176.6 (2)	O16—S14—C17—C18	−124.30 (18)
C6—C7—C8—C9	−5.6 (4)	O15—S14—C17—C18	8.3 (2)
C2—C7—C8—C13	−1.9 (2)	N1—S14—C17—C18	122.24 (18)
C6—C7—C8—C13	176.0 (2)	O16—S14—C17—C22	53.38 (19)
C13—C8—C9—C10	−5.5 (3)	O15—S14—C17—C22	−174.07 (17)
C7—C8—C9—C10	176.3 (2)	N1—S14—C17—C22	−60.09 (19)
C13—C8—C9—C25	175.0 (2)	C22—C17—C18—C19	−1.2 (3)
C7—C8—C9—C25	−3.2 (3)	S14—C17—C18—C19	176.40 (17)
C8—C9—C10—S24	−173.91 (17)	C17—C18—C19—C20	−0.2 (3)
C25—C9—C10—S24	5.6 (3)	C18—C19—C20—C21	2.1 (3)
C8—C9—C10—S11	5.9 (3)	C18—C19—C20—C23	−177.8 (2)
C25—C9—C10—S11	−174.60 (17)	C19—C20—C21—C22	−2.5 (3)
C9—C10—S11—C12	−2.1 (2)	C23—C20—C21—C22	177.3 (2)
S24—C10—S11—C12	177.70 (12)	C20—C21—C22—C17	1.2 (3)
C10—S11—C12—C13	−2.2 (2)	C18—C17—C22—C21	0.8 (3)
S11—C12—C13—N1	179.22 (17)	S14—C17—C22—C21	−176.89 (16)

### Hydrogen-bond geometry (Å, °)

**Table:**

*D*—H···*A*	*D*—H	H···*A*	*D*···*A*	*D*—H···*A*
C21—H21···O15^i^	0.95	2.50	3.387 (3)	155
C22—H22···O16^ii^	0.95	2.59	3.116 (3)	116

Symmetry codes: (i) *x*, *y*−1, *z*; (ii) −*x*+1/2, *y*−1/2, −*z*+1/2.
